# Energy-based devices in gynecology: the new frontier for the treatment of genitourinary syndrome of postmenopause?

**DOI:** 10.6061/clinics/2021/e3066

**Published:** 2021-06-23

**Authors:** José Maria Soares-Júnior, Maricy Tacla Alves Barbosa, Lana Maria Aguiar, Isadora Braga Seganfredo, Elsa Aida Gay de Pereyra, Nilson Roberto de Melo, Jorge Milhem Haddad, Edmund Chada Baracat

**Affiliations:** Disciplina de Ginecologia, Departamento de Obstetricia e Ginecologia, Hospital das Clinicas HCFMUSP, Faculdade de Medicina, Universidade de Sao Paulo, Sao Paulo, SP, BR

Genitourinary syndrome (GUS) is a very frequent disease after menopause and is caused by a sharp decline in estrogen levels (1). According to the North American Menopause Society, the current concept of the syndrome includes a set of signs observed during a physical examination and associated symptoms due to estrogen deficiency, involving the labia majora, labia minora, vestibule, clitoris, urethra, and bladder (2). Classical therapy consists of long-term use of topical estrogen (1,2). However, adherence to treatment is reduced over time, resulting in frequent relapses (1). New alternatives have also been sought for patients with contraindications to hormone therapy. Studies are being conducted using physical energies, such as laser, radio frequency, and ultrasonography, aimed at mitigating the consequences of hypoestrogenism in the lower genital tract (LGT) (3).

The US Food and Drug Administration (FDA) has licensed the CO_2_ laser systems for “incision, excision, ablation, vaporization, and coagulation of soft tissues in the body,” which have been used in specialties such as dermatology, otorhinolaryngology, neurosurgery, urology, and gynecology, including genitourinary surgery since 2010 (3). Specifically, energy-based devices (EBDs), such as the more commonly used radio frequency and laser, are authorized by the FDA for general indications of gynecological instrument use, including the destruction of abnormal cervical or vaginal tissue, condylomata, and precancerous lesions (3). Such indications have always been followed in gynecology (4).

In 2018, the FDA issued a warning about the indiscriminate utilization of the laser and other EBDs for cosmetic purposes, which could result in serious adverse events, such as vaginal burns, scarring, painful sexual intercourse, and recurrent/chronic pain (5). The warning was brought on mainly by the concern that the exaggerated expansion occurring in the United States and worldwide (6) has led many health professionals, physicians, and nonphysicians to use such devices without a precise indication, with the promise of genital rejuvenation.

In 2020, the North American Menopause Society issued a norm regarding the use of EBDs (4). Although they have not yet been approved by the FDA for use in GUS and are still considered an experimental treatment, several clinical studies have shown them to be effective in the treatment of the syndrome (7-11).

In general, the main benefits of EBD use in the LGT are as follows: a) thickening of the non-keratinized epithelium, b) increase in local angiogenesis, c) improvement in lubrification, d) normalization of the genital microbioma, e) decline in vaginal pH, f) development of new collagen fibers and restoration of vaginal architecture, improving elasticity and decreasing the frequency of microtraumas, and g) improvement in genitourinary symptoms (7-11). The latter was highlighted by the North American Menopause Society; however, it warned that training with an EBD should be provided with care and that inadequate use of the device poses a risk (4).

In a recent meta-analysis (12), the authors reported improvements in both genitourinary symptoms and their repercussions of urinary incontinence, quality of life, the vaginal health index, and sexuality. In all parameters, analysis of the treatments showed that the laser was beneficial for women in the control group (12). The authors also reported low rates of non-severe side effects. However, it should be noted that the number of participants was small, and follow-up was short (12); therefore, the long-term effects of such therapies cannot be assessed.

It is recommended that the indication of the procedure be conveyed in a clear and transparent way to the patients who should be instructed on the limitations of the method.

For the successful application of EBDs, it is also important and necessary that qualified health professionals receive adequate practical training before using the devices. Despite being promising, the techniques involving EBDs are still in the experimental stage; thus, further studies are necessary to evaluate the long-term benefits and risks for patients with GUS.

## AUTHOR CONTRIBUTIONS

Soares-Júnior JM, Seganfredo IB, Barbosa MTA and Baracat EC were responsible for the manuscript writing. Pereyra EAG and Aguiar LM were responsible for the literature search. Melo NR and Haddad JM were responsible for the manuscript review and corrections.
